# Chronic glucocorticoid exposure activates BK-NLRP1 signal involving in hippocampal neuron damage

**DOI:** 10.1186/s12974-017-0911-9

**Published:** 2017-07-21

**Authors:** Biqiong Zhang, Yaodong Zhang, Wenning Wu, Tanzhen Xu, Yanyan Yin, Junyan Zhang, Dake Huang, Weizu Li

**Affiliations:** Department of Pharmacology, Key Laboratory of Anti-inflammatory and Immunopharmacology, School of Basic Medical Sciences, Anhui Medical University, Hefei, 230032 China

**Keywords:** Glucocorticoids, NLRP1 inflammasome, BK channels, Neuroinflammation, Alzheimer’s disease

## Abstract

**Background:**

Neuroinflammation mediated by NLRP1 (nucleotide-binding oligomerization domain (NOD)-like receptor protein 1) inflammasome plays an important role in many neurological diseases such as Parkinson’s disease (PD) and Alzheimer’s disease (AD). Our previous studies showed that chronic glucocorticoid (GC) exposure increased brain inflammation via NLRP1 inflammasome and induce neurodegeneration. However, little is known about the mechanism of chronic GC exposure on NLRP1 inflammasome activation in hippocampal neurons.

**Methods:**

Hippocampal neurons damage was assessed by LDH kit and Hoechst 33258 staining. The expression of microtubule-associated protein 2 (MAP2), inflammasome complex protein (NLRP1, ASC and caspase-1), inflammatory cytokines (IL-1β), and large-conductance Ca^2+^ and voltage-activated K^+^ channel (BK channels) protein was detected by Western blot. The inflammatory cytokines (IL-1β and IL-18) were examined by ELISA kit. The mRNA levels of NLRP1, IL-1β, and BK were detected by real-time PCR. BK channel currents were recorded by whole-cell patch-clamp technology. Measurement of [K^+^]_i_ was performed by ion-selective electrode (ISE) technology.

**Results:**

Chronic dexamethasone (DEX) treatment significantly increased LDH release and neuronal apoptosis and decreased expression of MAP2. The mechanistic studies revealed that chronic DEX exposure significantly increased the expression of NLRP1, ASC, caspase-1, IL-1β, L-18, and BK protein and NLRP1, IL-1β and BK mRNA levels in hippocampal neurons. Further studies showed that DEX exposure results in the increase of BK channel currents, with the subsequent K^+^ efflux and a low concentration of intracellular K^+^, which involved in activation of NLRP1 inflammasome. Moreover, these effects of chronic DEX exposure could be blocked by specific BK channel inhibitor iberiotoxin (IbTx).

**Conclusion:**

Our findings suggest that chronic GC exposure may increase neuroinflammation via activation of BK-NLRP1 signal pathway and promote hippocampal neurons damage, which may be involved in the development and progression of AD.

## Background

Glucocorticoids (GCs) are the primary hormones released from the adrenal gland in response to stressful events. Stress increases circulating levels of endogenous GCs (cortisol in humans and corticosterone in rodents) [1], which in turn may induce neurodegenerative diseases, such as Alzheimer’s disease (AD) and depression vulnerability [2, 3]. Growing data showed that prolonged stress and chronic GC exposure produced abnormal behaviors in experimental animals and increased risk of psychiatric disorders in humans, for example, chronic stress plays an important role in the etiology of sporadic AD [4–7]. Furthermore, stress-level GCs are known to reduce hippocampal dendritic complexity [8, 9] and promote hippocampal neurons injury [10]. These studies suggest that chronic exposure to stress-level GCs results in neuronal injury and contributes to the development of neurodegenerative diseases, but the precise molecular and cellular mechanisms remain to be fully elucidated.

An emerging literature suggests that neuroinflammation plays an important role in many neurological diseases such as Parkinson’s disease (PD) and AD [11, 12]. GCs have been traditionally appreciated for their potent anti-inflammatory properties, but growing investigation has revealed that depending on the context and duration of exposure, GCs can increase some of the inflammatory responses they normally inhibit in central nervous system (CNS) [13–15]. Chronic GC exposure that would normally suppress inflammatory responses in the periphery instead lead to increased CNS inflammation in response to bacterial lipopolysaccharide (LPS) [16] and excitotoxin, particularly in GR-rich regions like the frontal cortex and hippocampus [17]. Several studies have also shown that chronic stress and GCs can modulate the immunophenotype of CNS macrophages and microglia [14, 18, 19], augment the microglial proinflammatory response to LPS [20, 21], and enhance the TNF-α-mediated increase of Toll-like receptor (TLR) [22]. These data demonstrate that chronic exposure to GCs primes microglia to proinflammatory stimuli and suggest that GCs may have proinflammatory action. However, it remains unclear whether chronic GC exposure has proinflammatory effects on hippocampal neurons.

Inflammasomes are multi-protein complexes that regulate the activity of caspase-1 and promote the maturation of inflammatory cytokines IL-1β and IL-18, which have been shown to play an important role in neuronal injury [23]. The nucleotide-binding oligomerization domain (NOD) like receptor protein 1 (NLRP1) inflammasome is the first to be discovered and is composed of NLRP-1, an adaptor known as apoptosis-associated speck-like protein containing a caspase-activating recruitment domain (ASC), and caspase-1 [24]. NLRP1 inflammasome was mainly expressed in neurons and implicated in the processes of AD and epilepsy [12, 25, 26]. It has been reported that chronic GC exposure increased the gene expression of NLRP3, Iba-1, MHCII, and NF-κBIα in a concentration-dependent manner in microglia [21]. Our latest study showed that chronic dexamethasone (DEX) treatment (21 and 28 days) induced significant neurodegeneration and activated NLRP1 inflammasome in the frontal cortex and hippocampus brain tissue [27]. However, the precise mechanisms of chronic GC exposure on the activation of NLRP1 inflammasome in hippocampal neurons remain to be fully elucidated.

Recently, the role of K^+^ in the activation of inflammasome is documented. Low intracellular K^+^ concentration ([K^+^]_i_) is a requirement for NLRP1 and NLRP3 inflammasome activation [28, 29]. In vitro, NLRP inflammasome assembly and caspase-1 recruitment occur spontaneously at [K^+^]_i_ below 90 mM, but is prevented at higher concentrations [30]. To induce NLRP3 activation, this mechanism of K^+^ ions depletion additionally requires an influx of Ca^2+^ through transient receptor potential (TRP) channels and activation of the TGF-β-activated kinase 1 (TAK1) [28]. Large-conductance Ca^2+^ and voltage-activated K^+^ channels (BK channels), which are gated by Ca^2+^ influx, contribute to action potential repolarization in neurons and play an important role in regulating neurotransmitter release [31]. Outward K^+^ currents through BK channels repolarize the cell and reduce excitability. Furthermore, BK channels are important for the K^+^ transport [32]. GCs have been shown to regulate BK channel sensitivity to phosphatase activity in pituitary-related cells. DEX, a synthetic glucocorticoid, reversibly increased the density BK current (*I*_K(Ca)_) in pituitary GH_3_ and AtT-20 cells [33]. However, to our knowledge, similar modulation of BK channels by GCs has not been shown in hippocampal neurons. And it is still unclear whether chronic GC exposure can induce the activation of NLRP1 inflammasome by regulating the BK channels.

DEX is a synthetic GC drug. The doses of DEX from 0.035 to 1 mg/kg were widely prescribed in clinic for treating many diseases [34, 35], while the doses from 0.5 to 80 mg/kg were widely used in animals to study the neurodegenerative diseases [36–38]. Our prior study showed that DEX (5 μM) exposure for 3 days significantly increased expression of NLRP1 inflammasome in hippocampal neurons [39]. In the present study, we further investigated the mechanisms of chronic DEX (5 μM) treatment on BK-NLRP1 inflammasome signal in hippocampal neurons. The study had the potential to contribute to a more complete understanding of the mechanisms by which GCs may involve in neurodegeneration and progression of AD.

## Methods

### Hippocampal neuron cultures and treatment

Primary hippocampal neurons were isolated from hippocampus of postnatal (0–24 h) Sprague Dawley rats via methods described previously [40, 41]. The primary neurons were maintained in Neurobasal medium with B27 supplements (Invitrogen, USA). Cells were plated onto poly-l-lysine (10 μg/ml)-coated 96-well culture plates (5 × 10^4^ cells/well) or glass coverslips in 24-well culture plates (1.5 × 10^5^ cells/well) or 6-well culture plates (1 × 10^7^ cells/well). The hippocampal neurons were cultured for 5 days before being treated with dexamethasone (DEX) (Sigma, USA) or iberiotoxin (IbTx) (Sigma, USA), the BK channel inhibitor. In prior studies, IbTx (0.2 μM) significantly decreased the peak amplitude of BK channel currents [42, 43]. The medium was replaced every 2 days. The hippocampal neurons were divided into four groups (control, DEX 5 μM-treated, IbTx 0.2 μM-treated, and DEX 5 μM + IbTx 0.2 μM-treated groups) or six groups (control 1-day, 3-day, and 5-day groups; DEX 5 μM-treated 1-day, 3-day, and 5-day groups).

### Animals and treatment

Male ICR mice (22–26 g) were housed under standard conditions and kept on a 12-h light/dark cycle with ad libitum access to food and water. These animals were randomly divided into eight groups: groups of control for 7, 14, 21, and 28 days and groups of DEX treatment for 7, 14, 21, and 28 days (*n* = 4). Animals in DEX treatment groups were treated with DEX (Sigma, USA) at 5 mg/kg/day (s.c.), while the mice in control groups were injected with normal saline (NS) with equal volume of alcohol. DEX solution was prepared by dissolving DEX in alcohol at a concentration of 500 mg/ml and diluted in normal saline at a concentration of 0.5 mg/ml. After DEX treatment for 7, 14, 21, and 28 days, the control group and DEX-treated group mice were euthanized; the brains were carefully removed. Half of the brain hippocampus tissues were used for immunoblot assays; the other half of brain hippocampus tissues were used for quantitative real-time PCR analysis.

### LDH release

To observe the effects of chronic DEX and IbTx exposure on hippocampal neuron injury, the activity of LDH released to the medium was determined after DEX or IbTx treatment for 3 days as described previously [43]. The activity of LDH was performed according to the protocols of LDH kit. Briefly, an aliquot of the culture supernatants was mixed with nicotinamide adenine dinucleotide (NAD) and lactate solution. Colorimetric absorbance was measured at 490 nm with a microplate reader (SPECTRAMAX 190, USA).

### Hoechst 33258 staining

To confirm the hippocampal neuron damage, the apoptosis rate of hippocampal neurons was evaluated by using Hoechst 33258 nuclear staining as described previously [40, 44]. For Hoechst 33258 staining, the hippocampal neurons were fixed with 4% paraformaldehyde after DEX or IbTx treatment for 3 days. The neurons were incubated with Hoechst 33258 (5 μg/ml, Zhongshan Golden Bridge Biotechnology Co.) for 15 min, washed three times with PBS, and mounted onto slides using anti-fade mounting medium (Beyotime Biotechnology Co.). Then, the neurons were examined by fluorescence microscopy (Olympus IX71) (Ex/Em: 352 nm/461 nm), and images were captured at 400 magnification. Morphologically, cells undergoing apoptosis appear smaller than normal and the nucleus appears condensed and deeply staining [45]. The percentage of neuronal apoptosis rate was determined in each culture.

### Immunofluorescence

The microtubule-associated protein 2 (MAP2) is a cytoskeletal protein localized in the neuronal dendritic compartment. The MAP2 is considered a marker of structural integrity because it is involved in morphological stabilization of dendritic processes [46]. The immunofluorescence was used to observe the expression of MAP2 after DEX or IbTx treatment for 5 days. For the immunofluorescence, the hippocampal neurons were fixed with 4% paraformaldehyde for 30 min at room temperature followed by three washes in PBS. Neurons were permeabilized with 0.25% Triton X-100 for 30 min and blocked with 1% BSA in PBS for 1 h. Then, the neurons were incubated with primary antibodies of mouse anti-MAP2 (1:200, Abcam) overnight at 4 °C. Secondary antibodies directed against mouse were conjugated to FITC (1:200, ZSGB-BIO). The stained cells of MAP2 were mounted using anti-fade medium. Then, slides were examined with confocal laser-scanning microscope (Leica Microsystems, Heidelberg, Germany).

### Immunoblot

Western blotting was performed as previously described [43]. (1) After DEX 5 mg/kg treatment for 7, 14, 21, and 28 days, the control and DEX-treated mice were euthanized and the total protein of hippocampus tissue was extracted. (2) After DEX or IbTx treatment for 3 or 5 days, the total protein of hippocampal neurons was extracted. (3) After DEX 5 μM treatment for 1, 3, and 5 days, the total protein of hippocampal neurons was extracted. All the proteins were stored at −80 °C for immunoblot assays. The protein concentration was determined by BCA Protein Assay Kit (Shanghai Sang on Bio-Tech). Equal amount of protein (40 μg) was separated by SDS-PAGE and transferred to polyvinylidene difluoride (PVDF) membranes. The membranes were blocked at room temperature for 1 h with 5% dry skim milk in tris-buffered saline containing 1% Tween-20 (TBS-T). Then, the membranes were reacted with antibody of MAP2 (1:500, Abcam), NLRP1 (1:500, Abcam), ASC (1:500, Bioworld), caspase-1 (1:500, Bioworld), IL-1β (1:500, Abcam), BK (1:500, Abcam), and β-actin (1:1000) overnight at 4 °C. Then, the membranes were extensively washed and incubated with IgG antibody conjugated to HRP (1:10,000) for 1 h. After extensive washes, the protein bands were detected by chemiluminescence reagents (ECL kit; Amersham Biosciences, Little Chalfont, UK). The Chemi Q4800 mini Imaging System (Shanghai Bioshine Technology) was used to visualize protein bands, and densitometry was performed with Image J software. The relative density of immunoreactive bands was normalized to the density of the corresponding bands of β-actin.

### The enzyme-linked immunosorbent assay (ELISA)

The supernatants were collected after incubation with DEX or IbTx treatment for 3 days. The ELISA kit was used for the quantitative determination of IL-1β and IL-18 (Cloud-Clone Corp.). IL-1β and IL-18 standards and samples were added to the wells of assay plates and incubated for 1 h at 37 °C. Blank wells were added with standard diluent. The horseradish peroxidase (HRP) conjugated reagent (100 μl) was added to each well for 1 h at 37 °C. Plates were washed four times with PBS, and chromogen solution (100 μl) was added to each well. The plates were gently mixed and incubated for 15 min at 37 °C in the dark. Then, stop solution (50 μl) was added to each well and examined the absorbance at 450 nm with a microplate reader (SPECTRAMAX 190, USA) within 15 min.

### Quantitative real-time PCR

For the PCR analysis, total RNA was extracted from hippocampus tissues and cultured hippocampal neurons with TRIzol reagent (Invitrogen Co., USA) according to the manufacturer’s instructions as described previously [27]. The first-strand cDNA was synthesized from total RNA with PrimeScript™ Reverse Transcriptase (Takara Bio) according to the manufacturer’s protocol. Quantitative real-time PCR analyses for mRNAs of NLRP1, IL-1β, BK, and β-actin were performed with SYBR®Premix Ex Taq™II RTPCR kits (Takara Bio). The mRNA level of β-actin was used as an internal control. The primers were constructed based on the published nucleotide sequences as follows: NLRP1 (XM 017314354.1, forward (2441–2460): 5-TGG CAC ATC CTA GGG AAA TC-3, reverse (2255–2236): 5-TCC TCA CGT GAC AGC AGA AC-3); IL-1β (LT 727137.1, forward (757–738): 5-CTG CTT CCA AAC CTT TGA CC-3, reverse (638–657): 5-AGC TTC TCC ACA GCC ACA AT-3); BK (XM 017315887.1, forward (3970–3989): 5-GGG ATG GTG GTT GTT ATG GT-3, reverse (4118–4099): 5-CTC GTA GGG AGG ATT GGT GA-3); β-actin (forward: 5-GAT TAC TGC TCT GGC TCC TAG C-3, reverse: 5-GAC TCA TCG TAC TCC TGC TTG C-3). PCR was performed at 95 °C for 10 min, followed by 40 cycles of amplification at 95 °C for 15 s, 60 °C for 30 s and 72 °C for 30 s with Real-time PCR System (ABI 7500, USA). The fluorescent signals were collected during extension phase, Ct values of the sample were calculated, and transcript levels were analyzed by 2^−ΔΔCt^ method. The PCR was repeated three times.

### Measurement of intracellular K^+^ concentration

Ion-selective electrode (ISE) technology is widely accepted as the method of choice for measuring potassium concentrations [47]. The c311 automatic biochemical analyzer (Roche Co.) was used to measure potassium by use of ISE technology. To observe the effects of DEX and IbTx treatment on [K^+^]_i_, the hippocampal neurons were divided into six groups in six-well culture plates: control, DEX 1 μM-treated, DEX 5 μM-treated, DEX 10 μM-treated, IbTx 0.2 μM-treated, and DEX 5 μM + IbTx 0.2 μM-treated groups. To avoid DEX-induced damage and reduction of neurons, the [K^+^]_i_ was examined after DEX or IbTx treatment for 2 h. Briefly, the hippocampal neurons were washed three times with PBS. Then, 0.5-ml double distilled water was added to the each well to lyses the hippocampal neurons. The lysates were collected and stored at −80 °C for measurement of [K^+^]_i_. The relative concentration of [K^+^]_i_ was normalized to the control group and repeated three times.

### Whole-cell patch-clamp recording

The whole-cell patch-clamp recording was executed as that described in previous reports with minor modification [31, 43]. For recording BK channel currents, the bath solution was composed of the following (in mM): 144 NaCl, 6 KCl, 1.2 MgCl_2_, 2 CaCl_2_, 10 HEPES, 10 D-glucose, and 5 4-AP, pH adjusted to 7.4 with NaOH. Glass pipettes were used with a resistance of 2–4 MΩ when filled with the following solution (in mM): 110 K-glutamine, 20 KCl, 3 Na_2_ATP, 0.1 EGTA, 3 MgCl_2_, 10 HEPES, and 10 D-glucose, pH adjusted to 7.2 with KOH. After establishing a whole-cell configuration, the adjustment of capacitance compensation and series resistance compensation was done before recording. The current signals were acquired at a sampling rate of 10 kHz and filtered at 3 kHz. Whole cell patch-clamp recordings were carried out using an EPC-10 amplifier (HEKA, Lambrecht, Germany) driven by Pulse/Pulse Fit software (HEKA, Southboro, Germany). Drug actions were measured after incubation for 5 min to reach steady-state conditions, which were judged by the amplitudes and time courses of currents remaining constant. All the recordings were made at room temperature (20–22 °C). All experiments were repeated three times using different batches of cells and at least three to four dishes with cells were used for recording in different batches of cells.

### Statistical analysis

Data are presented as mean ± SD. Statistical analyses were performed by using SPSS 17.0. Statistical differences were analyzed by one-way ANOVA and then subjected to between-group comparisons using the Bonferroni’s post hoc test. Differences were considered significant at a value of *P* < 0.05.

## Results

### Effects of chronic DEX and IbTx exposure on hippocampal neuron damage and apoptosis

**Fig. 1:**
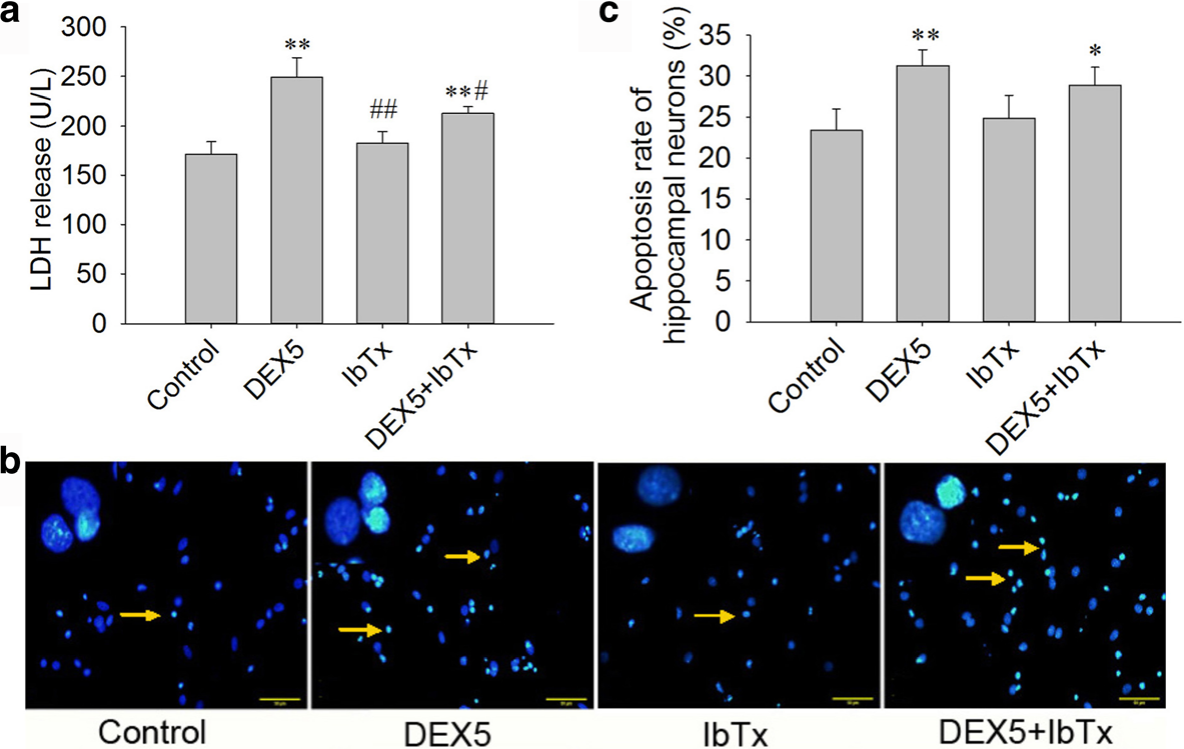
Effect of chronic DEX and IbTx treatment on hippocampal neurons damage and apoptosis. **a** The results of DEX and IbTx treatment for 3 days on LDH release in medium. **b** The results of DEX and IbTx treatment for 3 days on hippocampal neuron apoptosis (Hoechst 33258 staining, ×400). **c** The analysis of the percent of apoptosis in hippocampal neurons. Results are expressed as mean ± SD, LDH assay *n* = 3, Hoechst staining *n* = 4. *P < 0.05, **P < 0.01 compared to control group; ^#^ *P* < 0.05, ^##^ *P* < 0.01 compared to DEX 5 μM group

To observe the effects of chronic DEX and IbTx treatment on hippocampal neuron damage, the hippocampal neurons were treated with DEX (5 μM) or DEX (5 μM) + IbTx (0.2 μM) for 3 days. Then, the activity of LDH released in supernatant was detected. The results showed that DEX (5 μM) treatment for 3 days induced neuronal injury and significantly increased LDH release (Fig. 1a; *P* < 0.01). IbTx alone treated for 3 days had no significant effect on LDH release, but compared with DEX-treated group, IbTx significantly decreased the LDH release in the presence of DEX (Fig. 1a; *P* < 0.05).

We further examined the effects of chronic DEX and IbTx treatment on neuronal apoptosis by staining with Hoechst 33258. Hoechst 33258 can bind to chromatin, allowing fluorescent visualization of normal and condensed chromatin [45]. The results showed that there were few apoptotic neurons in control and IbTx-treated groups (Fig. 1b, c), while in DEX (5 μM)- and DEX (5 μM) + IbTx (0.2 μM)-treated groups, the apoptotic neurons significantly increased (Fig. 1b, c; *P* < 0.01, *P* < 0.05). Compared with DEX (5 μM)-treated group, IbTx had a trend to decrease neuronal apoptosis (Fig. 1b, c; *P* > 0.05). Our results suggest that chronic DEX exposure significantly accelerates the hippocampal neuron injury. And BK channel inhibiter, IbTx, has a protective effect on chronic DEX-induced neuronal damage.

### Effects of chronic DEX and IbTx exposure on expression of MAP2 in hippocampal neurons

**Fig. 2:**
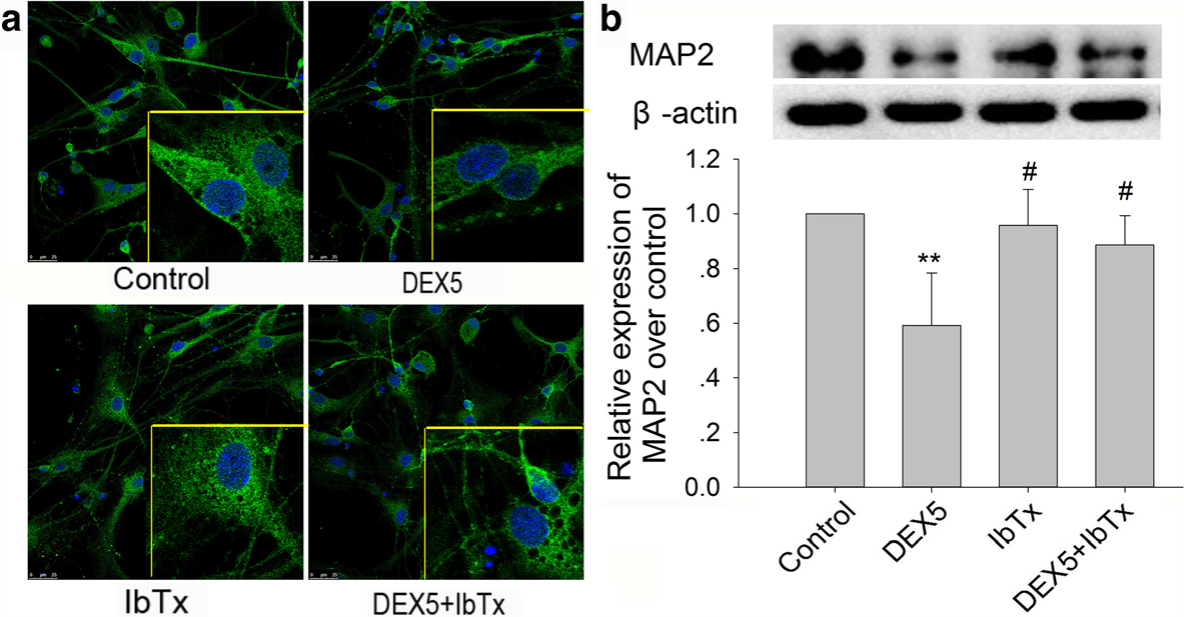
Effect of chronic DEX and IbTx treatment on MAP2 expression in hippocampal neurons. **a** The results of DEX and IbTx treatment for 5 days on MAP2 expression in hippocampal neurons (immunofluorescence, ×400). **b** The immunoblot results of DEX and IbTx treatment for 5 days on MAP2 expression and quantitative analysis of the expression of MAP2. Results are expressed as mean ± SD, immunofluorescence *n* = 3, immunoblot *n* = 4. **P < 0.01 compared to control group; ^#^ *P* < 0.05 compared to DEX 5 μM-treated group

The MAP2 is a cytoskeletal protein localized in the neuronal dendritic compartment. We further investigated the expression of MAP2 in the hippocampal neurons by immunofluorescence and immunoblot. The results showed that the expression of MAP2 was abundant in cytoplasm of hippocampal neurons in control and IbTx-treated group (Fig. 2). Compared with control group, DEX 5 μM treatment for 5 days significantly decreased MAP2 expression in the hippocampal neurons (Fig. 2; *P* < 0.01). Compared with DEX 5 μM-treated group, IbTx significantly increased the expression of MAP2, which reduced by chronic DEX treatment in hippocampal neurons (Fig. 2; *P* < 0.05).

### Effects of chronic DEX and IbTx exposure on expressions of NLRP1, ASC, caspase-1, IL-1β, and IL-18 in hippocampal neurons

**Fig. 3:**
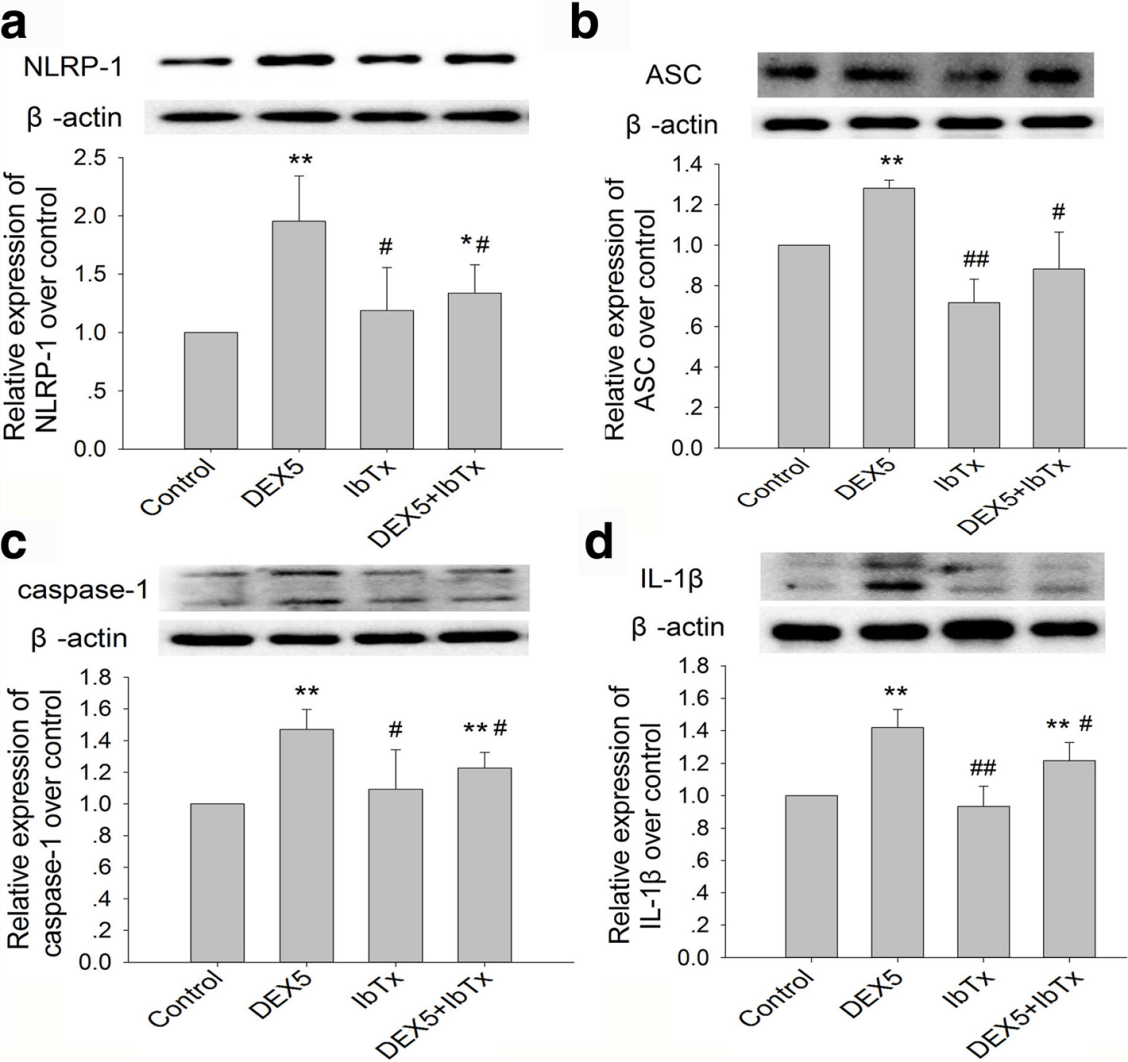
Effects of chronic DEX and IbTx treatment on expressions of NLRP1, ASC, caspase-1, and IL-1β (immunoblot). **a** The results of DEX and IbTx treatment for 3 days on expression of NLRP1. **b** The results of DEX and IbTx treatment for 3 days on expression of ASC. **c** The results of DEX and IbTx treatment for 3 d on expression of caspase-1. **d** The results of DEX and IbTx treatment for 3 days on expression of IL-1β. Results are expressed as mean ± SD, *n* = 4. *P < 0.05, **P < 0.01 compared to control group; ^#^ *P* < 0.05, ^##^ *P* < 0.01 compared to DEX 5 μM-treated group

**Fig. 4:**
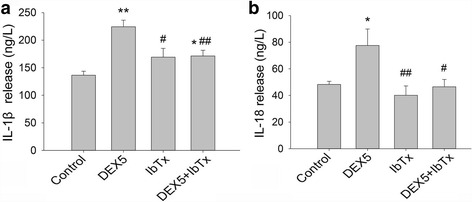
Effect of chronic DEX and IbTx treatment on the release of IL-1β and IL-18 in the supernatants (ELISA). **a** The results of DEX and IbTx treatment for 3 days on the release of IL-1β. **b** The results of DEX and IbTx treatment for 3 days on the release of IL-18. Results are expressed as mean ± SD, *n* = 4. *P < 0.05, **P < 0.01 compared to control group; ^#^ *P* < 0.05, ^##^ *P* < 0.01 compared to DEX 5 μM-treated group

To confirm whether NLRP1 inflammasome activation is involved in chronic DEX-induced hippocampal neurons damage, we investigated the effects of DEX and IbTx exposure on the expression of NLRP1, ASC, caspase-1, IL-1β, and IL-18 in hippocampal neurons or in the supernatant by immunoblot and ELISA. The immunoblot results showed that, compared with control group, DEX 5 μM treatment for 3 days significantly increased the expression of NLRP1, ASC, caspase-1, and IL-1β (Fig. 3a–d; *P* < 0.01), while compared with DEX 5 μM-treated group, IbTx significantly reduced the expression of NLRP1, ASC, caspase-1, and IL-1β in the hippocampal neurons which increased by chronic DEX treatment (Fig. 3a–d; *P* < 0.05). The ELISA results showed that, compared with control group, DEX 5 μM treatment for 3 days significantly increased the release of IL-1β and IL-18 in the supernatant of hippocampal neurons (Fig. 4a, b; *P* < 0.01 or *P* < 0.05), while compared with DEX 5 μM-treated group, IbTx significantly reduced the release of IL-1β and IL-18 which increased by chronic DEX treatment (Fig. 4a, b; *P* < 0.05). These data suggest that chronic DEX exposure might accelerate the activation of NLRP1 inflammasome and the BK channel inhibitor might decrease the activation of NLRP1 inflammasome activated by DEX exposure.

### Effects of chronic DEX and IbTx exposure on mRNA expression of NLRP1 and IL-1β in hippocampal neurons

**Fig. 5:**
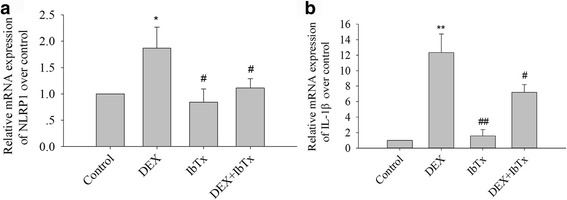
Effect of chronic DEX and IbTx treatment on mRNA expression of NLRP1and IL-1β in hippocampal neurons. **a** The results of DEX and IbTx treatment for 3 days on mRNA expression of NLRP1. **b** The results of DEX and IbTx treatment for 3 days on mRNA expression of IL-1β. Results are expressed as mean ± SD, *n* = 3. *P < 0.05, **P < 0.01 compared to control group; ^#^ *P* < 0.05, ^##^ *P* < 0.01 compared to DEX 5 μM-treated group

We further investigated the effects of DEX and IbTx exposure on mRNA expression of NLRP1and IL-1β in hippocampal neurons. The results showed that, compared with control group, DEX 5 μM treatment for 3 days significantly increased the mRNA expression of NLRP1and IL-1β in hippocampal neurons (Fig. 5a, b; *P* < 0.05 or *P* < 0.01), while compared with DEX 5 μM-treated group, IbTx significantly reduced the mRNA expression of NLRP1and IL-1β in hippocampal neurons (Fig. 5a, b; *P* < 0.05).

### Effects of chronic DEX exposure on the expression of BK mRNA and protein in hippocampus brain tissue in mice

**Fig. 6:**
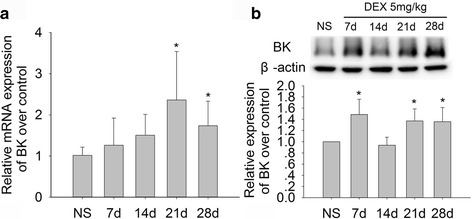
Effects of chronic DEX treatment on expression of BK mRNA and protein in hippocampus tissue in mice. **a** The results of DEX 5 mg/kg treatment for 7, 14, 21, and 28 days on expression of BK mRNA in hippocampus tissue (real-time PCR). **b** The results of DEX 5 mg/kg treatment for 7, 14, 21, and 28 days on expression of BK channel protein in hippocampus tissue (immunoblot). Results are expressed as mean ± SD, real-time PCR *n* = 3, immunoblot *n* = 4. *P < 0.05 compared to control group

To investigate whether BK channels involve in activation of NLRP1 inflammasome induced by chronic DEX exposure in hippocampal neurons, we detected the expression of BK mRNA and protein in the hippocampal tissues in mice by real-time PCR and immunoblot. The PCR results showed that, compared with the control group, DEX 5 mg/kg treatment for 7 and 14 days had a trend to increase the expression of BK mRNA, while DEX treatment for 21 and 28 days significantly increased the expression of BK mRNA in mice (Fig. 6a; *P* < 0.05). The immunoblot results showed that DEX 5 mg/kg treatment for 7, 21, and 28 days significantly increased the expression of BK channel protein (Fig. 6b; *P* < 0.05). Our results suggest that chronic GC exposure could significantly increase the expression of BK channel.

### Effects of chronic DEX exposure on the expression of BK channel protein in hippocampal neurons

**Fig. 7:**
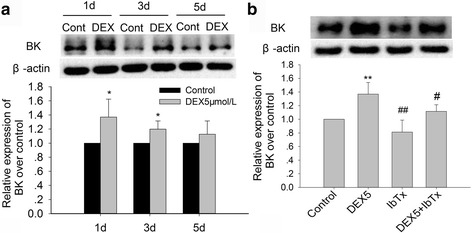
Effect of chronic DEX and IbTx treatment on the expression of BK in hippocampal neurons (immunoblot). **a** The results of DEX 5 μM treatment for 1, 3, and 5 days on expression of BK in hippocampal neurons. **b** The results of DEX and IbTx treatment for 3 days on the expression of BK in hippocampal neurons. Results are expressed as mean ± SD, *n* = 4. *P < 0.05, **P < 0.01 compared to control group; ^#^ *P* < 0.05, ^##^ *P* < 0.01 compared to DEX 5 μM-treated group

To confirm the effect of chronic GC exposure on BK channel expression in hippocampal neurons, we further investigated the expression of BK channel induced by chronic DEX and IbTx treatment in vitro. Firstly, we detected the effects of DEX 5 μM treatment for 1, 3, and 5 days on expression of BK channel. The results showed that, compared with control group, DEX treatment for 1 and 3 days significantly increased the expression of BK (Fig. 7a; *P* < 0.05). Secondly, we detected the effects of DEX 5 μM and IbTx treatment for 3 days on the expression of BK. The results showed that DEX 5 μM treatment for 3 days significantly increased the expression of BK (Fig. 7b; *P* < 0.01). While compared with DEX-treated group, IbTx treatment for 3 days significantly decreased the expression of BK which increased by DEX treatment (Fig. 7b; *P* < 0.05). Our results confirmed that chronic DEX could increase the expression of BK in hippocampal neurons, and IbTx, the BK channel inhibitor, could decrease the expression of BK in the presence of DEX.

### Effects of DEX and IbTx treatment on the [K^+^]_i_ and BK channel currents in hippocampal neurons

**Fig. 8:**
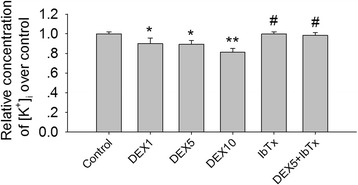
Effects of DEX and IbTx treatment on the [K^+^]_i_ in hippocampal neurons (Ion-selective electrode). The results showed that DEX (1, 5, and 10 μM) treatment for 2 h significantly decreased [K^+^]_i_ in hippocampal neurons. Compared with DEX 5 μM-treated group, IbTx significantly increased [K^+^]_i_ in presence of DEX. Results are expressed as mean ± SD, *n* = 3. **P < 0.01 compared to control group; ^#^ *P* < 0.05 compared to DEX 5 μM-treated group

Recently, the role of K^+^ in NLRP1 inflammasome activation is better interpreted [29, 48]. To determine whether K^+^ is involved in the activity of NLRP1 inflammasome induced by DEX treatment, we observed the change of [K^+^]_i_ induced by DEX and IbTx in hippocampal neurons. The results showed that DEX (1, 5, and 10 μM) treatment for 2 h significantly decreased [K^+^]_i_ in hippocampal neurons (Fig. 8; *P* < 0.01). Compared with DEX 5 μM-treated group, the BK channel inhibitor, IbTx, significantly increased [K^+^]_i_ which reduced by DEX 5 μM treatment (Fig. 8; *P* < 0.05).

**Fig. 9:**
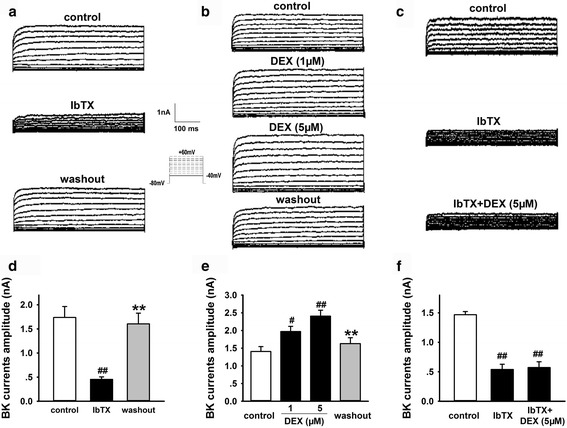
Effect of DEX and IbTx treatment on the BK currents in hippocampal neurons (whole-cell patch-clamp technology). **a** Representative traces of potassium currents induced by BK channel inhibitor IbTx. **b** Representative traces of potassium currents induced by DEX. **c** Representative traces of potassium currents induced by DEX and IbTx. **d** Statistical results showing recorded potassium currents were markedly inhibited by specific BK channel inhibitor IbTx. **e** Statistical results showing DEX increased BK currents. **f** Statistical results showing DEX failed to increase BK currents in the presence of IbTx. Results are expressed as mean ± SD, *n* = 5 or *n* = 4. ^#^ *P* < 0.05, ^##^ *P* < 0.01 compared to control group; **P < 0.01compared to DEX- or IbTx-treated group

BK channels are involved in cell excitability and neurotransmitter release in the CNS. Additionally, BK channels are also important for K^+^ transport [32]. To investigate whether BK channels contribute to the decrease of [K^+^]_i_ induced by DEX treatment, we recorded BK channel currents under DEX and IbTx stimuli by whole-cell patch-clamp technology. As shown in Fig. 9a, BK channel currents were elicited as described in our previous reports by applying 11 depolarizing pulses from −40 to +60 mV for 500 ms with a 10 mV increment from a holding potential of −80 mV [43]. To confirm whether the recorded currents were mediated by BK channels, the BK channel inhibitor IbTx was used. The results showed that IbTx (0.2 μM) markedly decreased the peak amplitude of recorded currents by 73.55 ± 4.70% (Fig. 9a, d; *n* = 5, *P* < 0.05), suggesting that recorded currents were carried by BK channels. Furthermore, DEX 1 μM and 5 μM significantly increased BK currents in hippocampal neurons (Fig. 9b, e; *n* = 5, *P* < 0.05, and *P* < 0.01). After washout, BK currents returned to the control level (Fig. 9b). While DEX 5 μM failed to increase BK currents in the presence of IbTx (0.2 μM) (Fig. 9c; *n* = 4, *P* < 0.01), indicating that the current potentiation induced by DEX is sensitive to IbTx. These data suggest that BK channels contribute to the effect of DEX treatment on [K^+^]_i_ in hippocampal neurons.

## Discussion

Chronic stress has been reported to be associated with many neurodegenerative diseases, such as depression, AD, and PD [2, 4, 49]. The chronic stress-induced neurodegenerative diseases are an outcome of different mechanisms, such as central neurotransmitters, neurohormonal factors, free radical generation, particularly the dysfunction of hypothalamic-pituitary-adrenal (HPA) axis [50, 51]. GCs are the primary hormones released from the adrenal gland in response to stressful events. It has been reported that the physiological plasma corticosterone concentration range in rats is roughly between 20 and 50 nM, while the stress levels of this hormone are considered to be from 100 to 200 nM or even higher [52]. Growing data suggest that high level of plasma GCs may be an important cause of chronic stress-induced neurodegeneration. In prior studies, hippocampal microglia isolated from chronic GC-exposed animals showed a potentiated response to LPS, which demonstrates that chronic GC exposure primes microglia to pro-inflammatory stimuli [18, 21]. Our prior study showed that chronic DEX exposure significantly activated the NLRP1 inflammasome and induced neuronal damage in hippocampal neurons [39]. In the current study, we demonstrate that NLRP1 inflammasome is activated by chronic DEX treatment and BK channel K^+^ signal mediates chronic DEX exposure-induced NLRP1 inflammasome activation, which is accountable for chronic GCs induced hippocampal neurons injury.

It has been reported that low [K^+^]_i_ (below 90 mM) could activate NLRP1 inflammasome in immune cells [30]. Also, valinomycin-triggered K^+^ efflux activates caspase-1 and increases IL-1β secretion in cultured spinal cord neurons [53]. Thus, [K^+^]_i_ may be a critical element in the activation of NLRP1 inflammasome. BK channels, which are gated by Ca^2+^ and voltage, contribute to action potential repolarization in neurons and play an important role in regulating [K^+^]_i_ [32]. GCs have been shown to regulate BK channel sensitivity to phosphatase activity in pituitary-related cells. Dexamethasone reversibly increases the density of BK current in pituitary GH_3_ and AtT-20 cells [33]. At present, whether chronic GC exposure can mediate NLRP1 inflammasome activation by increasing BK channel function is still unclear. We hypothesize that chronic GC exposure may upregulate the expression of BK channel, increase K^+^ efflux, and lead to low [K^+^]_i_, which mediates the activation of NLRP1 inflammasome and induces hippocampal neurons injury.

To confirm our hypothesis, we first investigated the effects of chronic DEX and BK channel inhibitor IbTx treatment on hippocampal neurons injury in vitro. We found that DEX treatment significantly increased LDH release in supernatant and accelerated hippocampal neuron apoptosis, while DEX failed to increase LDH release in the presence of IbTx. The results suggest that the hippocampal neuron injury induced by chronic DEX exposure is sensitive to IbTx. Meanwhile, we found that IbTx had a trend to decrease neuronal apoptosis (*P* > 0.05). It is unclear what is responsible for the phenomenon. We think that chronic DEX exposure may lead to neuronal damage and apoptosis and IbTx may mainly inhibit the neuronal damage, such as inflammatory injury, rather than apoptosis. Further efforts will be made to clarify the precise mechanism in future research. MAP2, a cytoskeletal protein localized in the neuronal dendritic compartment, is considered a marker of structural integrity because it is involved in morphological stabilization of dendritic processes [46]. The expression of MAP2 coincides with dendritic outgrowth, branching, and postlesion dendritic remodeling, suggesting that MAP2 plays a crucial role in plasticity of neurons [54]. In the present study, we found that chronic DEX treatment for 5 days significantly decreased the expression of MAP2 in hippocampal neurons. IbTx could increase the expression of MAP2 which reduced by chronic DEX treatment. These data suggest that chronic GC exposure can induce hippocampal neurons injury and the mechanism may be related to the regulation of BK-NLRP1 inflammasome signal.

The inflammasomes are multiprotein complexes that are responsible for the formation of proinflammatory molecules. The NLRP1 inflammasome is first characterized as a member of the NLRP family, whose activation can generate a functional caspase-1-containing inflammasome to cleave the precursors of IL-1β and IL-18 to yield active cytokines [26]. NLRP1 inflammasome is also highly expressed in pyramidal neurons of the brain [55] and has a key role in the pathogenesis of neurological disorders [12, 56]. The NLRP1 inflammasome consists of NLRP1, ASC, and caspase-1 [57]. The ASC is an important component of the inflammasomes, which connects the NLRPs to caspase-1 [58]. Caspase-1 is a critical modulator for maturation from pro-IL-1β and pro-IL-18 to their biologically active forms of IL-1β and IL-18 [59]. Therefore, the inflammasome is necessary for caspase-1 activation and IL-1β and IL-18 release and participates in the amplification of the inflammatory response and the promotion of cell death [60, 61]. Our earlier results showed that DEX 5 μM treatment for 3 days significantly activated NLRP1 inflammasome and increase the release of IL-1β and IL-18 in the supernatant. GC receptor antagonist RU486 could significantly decrease the expression of NLRP1, caspase-1, and IL-1β in hippocampal neurons and reduce the release of IL-1β and IL-18 [39]. However, whether GCs can activate the NLRP1 inflammasome by modulating BK channels remains unknown. In the present study, the results showed that DEX treatment for 3 days significantly increased the release of IL-1β and IL-18 in supernatant and increased the expression of NLRP1, ASC, caspase-1, and IL-1β in hippocampal neurons, while IbTx could inhibit DEX-induced activation of NLRP1 inflammasome in hippocampal neurons. These results suggest that chronic GC exposure can induce NLRP1 inflammasome activation and BK channel may be involved in regulation of NLRP1 inflammasome induced by chronic DEX treatment.

The BK channel is ubiquitously expressed in the nervous system and plays an important modulator of neuronal function. It has been reported that BK channel could modulate neuronal excitability, firing rate, and neurotransmitter release [62–64]. The ability of GCs to both reduce neuronal firing rate in celiac ganglion cells and enhance firing rate in cardiovascular neurons located in the rostral ventrolateral medulla [65, 66] shows the importance of rapid steroid modulation in neuronal excitability. Recently, the acute application of DEX has been shown to increase BK channel activity in pituitary GH3 and AtT-20 cells and reduce the firing of action potentials in GH3 cells [33]. Moreover, GCs could facilitate BK activation in adrenal chromaffin cells and promoting rapid action potential repolarization [67]. Similar effects of GCs on pituitary corticotrope and somatotrope like cell lines have also been reported [33]. However, modulation of BK channels by GCs in hippocampal neurons has not been fully elucidated. Whether GCs modulate BK channels and involve in NLRP1 inflammasome in hippocampal neurons is not yet known.

Low [K^+^]_i_ is a potent activator for the NALP1 inflammasome, which then stimulates caspase-1 to cleave the proforms of IL-1ß and IL-18 cytokines [29]. Our prior study showed that DEX (5 mg/kg) treatment for 28 days significantly increased the expression of NLRP1 inflammasome and induced hippocampal neuronal damage [27]. To confirm whether BK channels involve in chronic DEX exposure induced NLRP1 inflammasome activation, we further detected the effects of chronic DEX treatment on expression of BK channel in vivo and in vitro. The results showed that DEX (5 mg/kg) treatment for 28 days significantly increased the expression of BK mRNA and protein in hippocampus tissue in mice. Meanwhile, we found that DEX (5 μM) treatment for 3 days significantly increased the expression of BK channels, but failed to increase the BK channels expression in the presence of IbTx (0.2 μM) in hippocampal neurons. The results suggest that chronic DEX can upregulate expression of BK channel via gene effects and may be involved in activation of NLRP1 inflammasome in hippocampal neurons. It is still unknown whether changes in BK activity are correlated with changes in [K^+^]_i_. To confirm whether DEX exposure can lead to low [K^+^]_i_ by activating BK channel in hippocampal neurons, we detected the acute effect of DEX and IbTx treatment for 2 h on [K^+^]_i_ in hippocampal neurons in vitro. We found that DEX treatment for 2 h significantly decreased [K^+^]_i_ in hippocampal neurons. The BK channel inhibitor IbTx could significantly increase [K^+^]_i_ in hippocampal neurons. The results suggest that DEX may decrease [K^+^]_i_ by activating BK channel via non gene effects.

Furthermore, it has been reported that physiologically relevant concentrations of GCs facilitate gating of BK channels in HEK-293 cells, within 10 s of application to cell-free inside-out patches and under whole cell conditions [68]. Therefore, we proposed that DEX might increase BK channel currents, which contribute to the lower [K^+^]_i_ and the activation of NLRP1 inflammasome in hippocampal neurons. We further detected the acute effect of DEX incubation for 5 min on BK channel currents in hippocampal neurons. The results showed that extracellular DEX (1, 5 μM) treatment significantly increased the BK channel currents and IbTx, the BK channel inhibitor, significantly reduced the effect. These data suggest that GC acute exposure (5 min) can activate the BK channel, which may involve in the lower [K^+^]_i_ induced by DEX sustained exposure (2 h).

## Conclusions

**Fig. 10:**
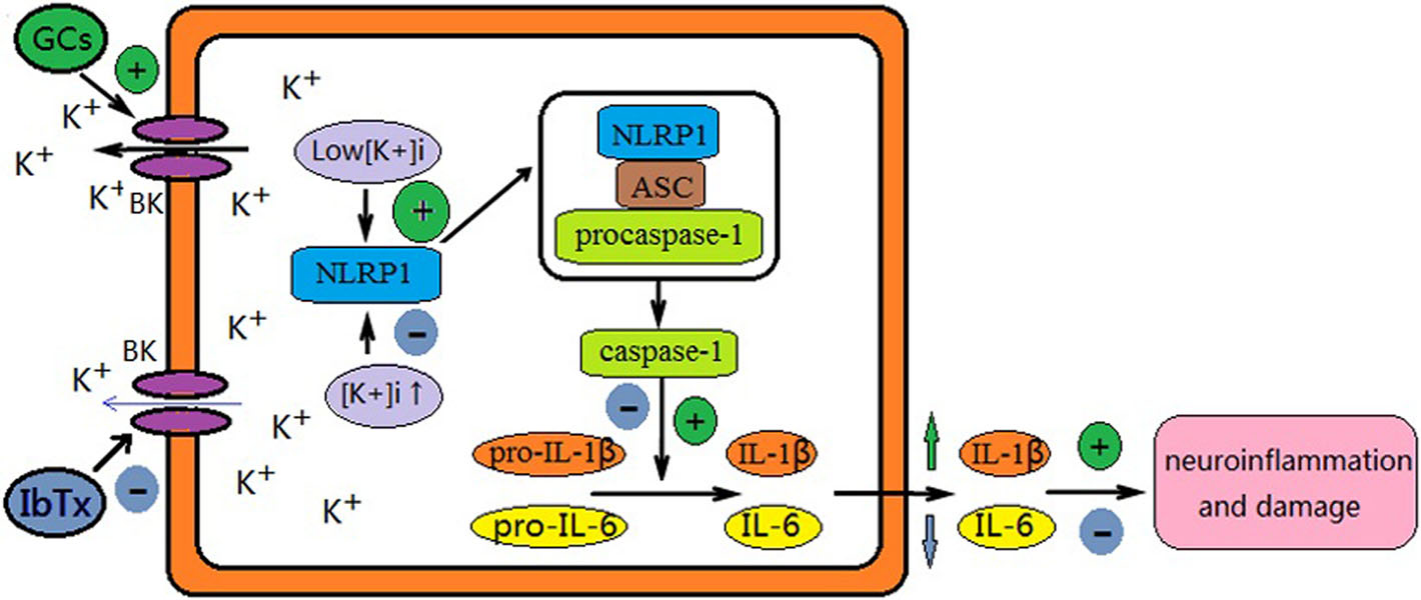
The scheme of the chronic GC exposure increases NLRP1 inflammasome via activation of BK channel. Chronic GC exposure activates NLRP1 inflammasome by upregulation and activation of BK channel and leading to low [K^+^]_i_ in the hippocampal neurons. BK channel inhibitor IbTx inhibits activation of NLRP1 inflammasome by blocking BK channel and increases [K^+^]_i_, which decreased by DEX treatment

Overall, the role of GCs on hippocampal neurons is complex. Chronic DEX exposure can induce neurodegeneration and accelerate NLRP1 inflammasome activation by modulating BK channel in hippocampal neurons. The mechanism of GCs-activated NLRP1 inflammasome in hippocampal neurons may be the result of the combination of gene effects and non-gene effects. Acute DEX exposure may activate BK channel and induce low [K^+^]_i_ via non gene effects, and chronic DEX exposure may upregulate expression of BK channel protein via gene effects, which may accelerate NLRP1 inflammasome activation and induces neurodegeneration in hippocampal neurons (Fig. 10). Our findings provide support for the hypothesis that chronic GC exposure may increase neuroinflammation via activation of BK-NLRP1 signal pathway and promote hippocampal neuronal damage. However, the study provided an experimental basis for chronic GC exposure on activation of BK-NLRP1 signal pathway. Other related mechanisms underlying the proinflammatory effects of GCs warrant further investigations.

## Abbreviations

AD: Alzheimer’s disease
ASC: Apoptosis-associated speck-like protein containing a caspase-activating recruitment domain
BK channels: Large-conductance Ca^2+^ and voltage-activated K^+^ channels
DEX: Dexamethasone
ELISA: Enzyme-linked immunosorbent assay
GCs: Glucocorticoids
IbTx: Iberiotoxin
IL-18: Interleukin-18
IL-1β: Interleukin-1β
LDH: Lactate dehydrogenase
MAP2: Microtubule-associated protein 2
NLRP1: Nucleotide-binding oligomerization domain (NOD)-like receptor protein 1
PD: Parkinson’s disease
